# Association of epigenetics of the *PDK4* gene in skeletal muscle and peripheral blood with exercise therapy following artificial knee arthroplasty

**DOI:** 10.1186/s40101-020-00216-y

**Published:** 2020-03-26

**Authors:** Tomohiro Kamo, Satoshi Kurose, Hiroshi Ohno, Minoru Murata, Takanori Saito, Yutaka Kimura

**Affiliations:** 1 Department of Health Science, Kansai Medical University, 2-5-1 Shinmachi, Hirakata, Osaka 573-1010 Japan; 2 Department of Orthopaedic Surgery, Kansai Medical University Hospital, 2-5-1 Shinmachi, Hirakata, Osaka 573-1010 Japan

**Keywords:** *PDK4* gene, Skeletal muscle, Peripheral blood, Promoter region, Pyrosequencing

## Abstract

**Background:**

Although exercise is a standard treatment for postoperative osteoarthritis, interindividual differences have been reported. Epigenetic modification (DNA methylation), a factor causing interindividual differences, is altered by the environment and may affect all tissues. Performing a tissue biopsy to investigate methylation of skeletal muscle fat metabolism genes is invasive, and less invasive and convenient alternatives such as blood testing are desired. However, the relationship between tissue and blood is still unclear. Here, we examined the relationship between DNA methylation of the *PDK4* gene in skeletal muscle and peripheral blood.

**Patients and methods:**

Five patients who underwent artificial knee arthroplasty between April 2017 and June 2018 at Kansai Medical University Hospital were included (2 men and 3 women; average age, 75.2 years; body mass index, 26.1 kg/m^2^). We measured the body composition of the patients using dual-energy X-ray absorptiometry. Peripheral blood was collected at the time of hospitalization and 5 months after surgery; skeletal muscles were collected at the time of surgery and 5 months after surgery. Rehabilitation was performed according to the clinical procedure for 3 months after surgery. Patients performed resistance training and aerobic exercise using an ergometer for 20 min twice a week. Biopsy samples were treated with bisulfite after DNA extraction, and the methylation rate was calculated at different CpG islands downstream from the transcription initiation codon of the *PDK4* gene.

**Results:**

No significant change in body composition was observed before and after postoperative exercise therapy, and no significant change was noted in the methylation at each position in the promoter region of *PDK4* in the skeletal muscle and peripheral blood. However, changes in the methylation rate at CpG1 in peripheral blood significantly correlated with those in skeletal muscle (*P* = 0.037). Furthermore, the amount of change in the methylation rate of CpG1 in the skeletal muscle was significantly correlated (*P* = 0.037) with the average methylation rate at the promoter region in peripheral blood.

**Conclusions:**

Methylation rates at CpG1 in the skeletal muscle and peripheral blood were significantly correlated, suggesting that skeletal muscle methylation could be analyzed via peripheral blood rather than skeletal muscle biopsy.

## Background

Osteoarthritis of the knee (OA knee) is a chronic and degenerative disease of the tissues of the knee joint that negatively affects the quality of daily life of elderly people because of pain during movement and a limited range of motion [1]. Moreover, body weight and fat mass often increase in OA knee patients. To treat and prevent OA knee, weight loss through diet and exercise is important; however, there are interindividual differences in the effectiveness of these measures [2, 3]. Genetic and epigenetic factors govern these interindividual differences in training effects. Genetic factors are the direct results of gene expression [4], whereas epigenetic factors regulate gene expression through addition or removal of methyl groups from genes or modification of histones in response to environmental factors [5, 6]. “Epigenetics” is a concept proposed by Waddington in 1956 [7] that involves DNA modification that controls and maintains gene expression without changes in the DNA sequence itself. Specifically, when a methyl group is added to DNA by the action of DNA methyltransferase, the respective transcription factor cannot bind to the promoter region, and transcription is suppressed. When the methyl group is removed, the transcription factor can bind to the promoter region and promote transcription [8, 9]. In this system, the type and amount of translated protein are regulated. Even though all cells have the same original base sequence, it is thought that gene expression is highly tissue specific, which enables cells to mature differentially and perform different functions in each organ [10]. Epigenetic modifications depend on the environment and may affect all tissues of the body [11].

The pyruvate dehydrogenase kinase 4 (*PDK4*) gene is associated with lipid metabolism in the skeletal muscle [12]. *PDK4* mediates the inactivation of pyruvate dehydrogenase complex (PDC) via phosphorylation. Inactivation of PDC inhibits the conversion from pyruvic acid to acetyl-CoA, thereby shifting the energy substrate utilization from carbohydrate to lipid [13, 14]. DNA methylation in the skeletal muscle plays an important role in gene regulation that depends on CpG methylation in promoter regions [15]. Moreover, hypomethylation of DNA occurs in the CpG island in the promoter region of the *PDK4* gene following exercise load in healthy people; this hypomethylation is a short-term effect that improves metabolism [16–19].

We previously investigated the changes in body composition and evaluated the methylation rate of the *PDK4* gene using next-generation sequencing (NGS) by performing exercise therapy in human [20]. We found that the exercise-mediated changes in body weight and body fat were associated with changes in the methylation rate of the promoter region and total CpG island [20]. However, NGS is an expensive analysis method and is difficult to perform [21]. Therefore, it is not suitable for clinical application at this point. If the analysis is limited to the promoter region, pyrosequencing would be more cost-effective and clinically feasible than NGS. In addition, it remains unclear whether the observed methylation of the *PDK4* gene in skeletal muscle tissue matches that in peripheral blood. If the relationship can be identified, it may not be necessary to invasively collect skeletal muscle tissue; instead, evaluation could be easily performed using peripheral blood. Thus, the current study aimed to calculate the methylation rate using pyrosequencing at each position of the promoter region of the *PDK4* gene and to verify the relevance of DNA methylation of the *PDK4* gene in skeletal muscle tissue compared with that in peripheral blood.

## Methods

### Patients

The enrolled patients visited the Department of Orthopedics Clinic at Kansai Medical University Hospital and underwent artificial knee arthroplasty between April 2017 and the end of June 2018 for osteoarthritis and osteonecrosis. There were five patients (5 knees), comprising two males and three females with an average age of 75.2 years and an average body mass index of 26.1 kg/m^2^ (Table 1). Exclusion criteria were rheumatoid arthritis (RA), mental disorders including dementia, inability to tolerate the exercise regimen, or a history of difficulty performing exercise therapy. This study was performed in accordance with the Declaration of Helsinki, and all procedures were approved by the Ethics Committee of the Kansai Medical University (Approval no. 2016709, date of approval: December 26, 2016). Written informed consent was obtained from all subjects prior to the start of the study.

**Table 1:** Patient characteristics and body compositions determined by dual-energy X-ray absorptiometry

Patient no.	Age (years)	Sex	BMI (kg/cm^2^)	Pre-TW (kg)	Pre-TFM (kg)	Pre-TLFM (kg)	Post-TW (kg)	Post-TFM (kg)	Post-TLFM (kg)
1	74	F	29.1	69.6	31.0	36.5	67.3	28.9	36.4
2	72	F	24.2	52	18.9	31.4	51.4	18.8	30.9
3	73	F	25.3	53.9	17.7	34.3	54.8	17.1	35.9
4	80	M	26.3	78.2	18.8	55.9	74.7	15.3	56.2
5	77	M	25.9	70.4	19.7	48.3	67.8	20.0	45.3

*Pre* preoperative, *Post* 5 months postoperative, *TW* total body weight, *BMI* body mass index, *TFM* total body fat mass, *TLFM* total lean body fat mass

### Body composition

The body composition of all patients was measured, including total body weight, total body fat mass, and total lean fat mass, using the Lunar Prodigy Advance dual-energy X-ray absorptiometry (DXA) system version 13.6 (GE Healthcare Co., Ltd., Waukesha, WI, USA) at the time of hospitalization and 5 months after surgery.

### Peripheral blood collection

Peripheral blood (20 cc) was collected from the cephalic vein of the elbow before and 5 months after orthopedic surgery at the time of the blood sampling test. Whole blood was stored in a −80 °C freezer.

### Muscle biopsy

Muscle biopsy of the affected limb was performed using a midvastus approach during surgery. Approximately 1 × 1 cm^2^ of the vastus medialis muscle was collected, frozen in liquid nitrogen, and stored in a freezer at −80 °C. At 5 months after surgery, the vastus medialis muscle was visualized under echo guidance at the same site where the biopsy specimen was collected during surgery. After local anesthesia with 1% Xylocaine® (Astra-Zeneca, Mölndal, Sweden), a needle biopsy was performed on each affected knee with a 14G biopsy needle (Medicon, Seoul, Korea). The obtained sample was also frozen in liquid nitrogen and stored in a freezer at −80 °C.

### Postoperative exercise therapy

Postoperative rehabilitative exercise therapy was performed according to the post-TKA/UKA protocol of our institution until 3 months after surgery. After surgery, continuous passive motion was performed until the knee joint movement exceeded 120°. Passive joint movement range exercises, lower limb strength training using ~3 metabolic equivalents (METs), and walking training for 10 min were carried out in the presence of a physical therapist. From the third month after surgery, patients performed resistance training, mainly involving the quadriceps muscle using ~3 METs, and an aerobic exercise using a bicycle ergometer at 40–50% strength according to the Karvonen method calculated from the predicted maximum heart rate. This exercise was performed for 20 min twice a week [22].

### Sodium bisulfite modification for DNA methylation analysis

Skeletal muscle samples were obtained from the affected knees using a QlAamp DNA Mini Kit (Qiagen, Hilden, Germany). Bisulfite-modified gDNA was prepared using the EZ DNA Methylation-Lightning™ kit (Zymo Research, Irvine, CA, USA) according to the manufacturer’s instructions. The bisulfite reaction was carried out using 200 ng gDNA (adjusted to a volume of 20 μl with sterile water) and 130 μl of CT Conversion Reagent. The sample tubes were placed in a thermal cycler (Thermo Scientific, Waltham, MA, USA) and subjected to the following conditions: 8 min at 98 °C, 60 min at 54 °C, and storage at 4 °C for up to 20 h. The DNA was then purified using reagents contained in the EZ DNA Methylation-Lightning™ kit (Zymo Research) according to the manufacturer’s instructions. The converted gDNA was eluted using 20 μl of M-Elution Buffer. DNA samples were finally stored at −20 °C until further use.

### Pyrosequencing analysis

We used the bisulfite pyrosequencing method for methylation analyses of the *PDK* gene. Each primer was designed using Pyrosequencing Assay Design Software v2.0 (Qiagen). PCR amplification was performed on a 76-base pair region at the −263 position of the *PDK4* gene using forward (5'–TTGGGATTAGGGGGGAGAG–3') and reverse (5'–biotin- CAAATTCAAATCTTCCCACCAA–3') primers. Each PCR mix contained 20 ng or more gDNA, PCR premixture (Enzynomics, Daejeon, Korea), 1 μl of 10 pmol/μl Primer-S, and 1 μl of 10 pmol/μl biotinylated-Primer-As. The amplification was carried out according to the general guidelines suggested for pyrosequencing: denaturing at 95 °C for 10 min, followed by 45 cycles at 95 °C for 30 s, 58 °C for 30 s, and 72 °C for 30 s, and a final extension at 72 °C for 5 min. The PCR reaction (2 μl) was confirmed by electrophoresis in a 2% agarose gel and visualized by ethidium bromide staining.

ssDNA templates were obtained with the assistance of the PyroMark ID Vacuum Prep Workstation (Qiagen) according to manufacturer’s instructions. Briefly, 16–18 μl of the PCR product was immobilized on streptavidin-coated Sepharose® HP beads (Amersham Biosciences, Uppsala, Sweden) and processed to obtain single-stranded DNA. This DNA template was then incubated with 15 pmol of the respective sequencing primer (5'–GATTAGGGGGGAGAGT–3') on the PyroMark ID heat-block at 80 °C for 2 min. Pyrosequencing was performed on a PyroMark ID system with the Pyro Gold reagents kit (Qiagen) according to the manufacturer’s instructions without further optimization.

### Statistical analysis

All data are expressed as mean ± standard deviation. We compared preoperative and 5-month postoperative measures using the Wilcoxon signed-rank test, including body composition (total weight, total lean mass, and total fat mass) and methylation in the *PDK4* gene in the skeletal muscle and peripheral blood; each position was expressed as 56 bp (CpG1), 76 bp (CpG2), 80 bp (CpG3), 83 bp (CpG4), and 86 bp (CpG5) downstream from the transcription initiation codon of the CpG island in the promoter region. Then, the methylation rate of each position was calculated and compared using the Wilcoxon signed-rank test. In addition, the relationship between age, the change in body composition, and the change in the *PDK4* methylation rate on the CpG island in peripheral blood and the skeletal muscle before and after surgery was evaluated using Spearman’s rank correlation coefficient. All statistical analyses were performed using SPSS software version 25.0 for Mac OS (IBM Corp., Armonk, NY, USA). *P* < 0.05 was considered statistically significant in all analyses.

## Results

### Changes in body composition

Analysis after surgery indicated that the total body weight had changed from 64.8 ± 11.4 kg to 63.2 ± 9.7 kg (*P* = 0.14), total body fat mass changed from 21.2 ± 5.5 kg to 20.0 ± 5.3 kg (*P* = 0.14), and total lean body fat mass changed from 41.3 ± 10.4 kg to 41.0 ± 10.0 kg (*P* = 0.69), as measured by DXA. These three parameters were not significantly different between before and 5 months after surgery.

### *PDK4* methylation results

**Fig. 1:**
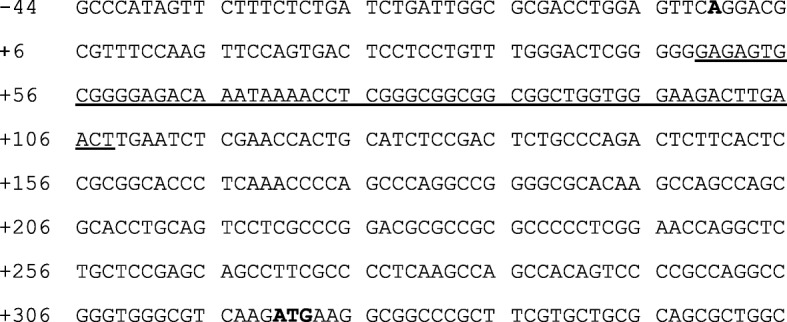
The CpG island region of the *PDK4* gene. The circled C represents cytosine reacted with bisulfite. The circled C is defined as CpG1, CpG2, CpG3, and CpG4, in that order. **A** is the transcription initiation codon. **ATG** is the translation initiation codon. The underlined portion is the promoter region of the *PDK4* gene

The CpG island region of *PDK4* is shown in Fig. 1. A total of 77 CpG sites were sequenced. The promoter region of the *PDK4* gene from the Swiss Institute of Bioinformatics (Lausanne, Switzerland) comprises 60 bp (5'–GAGAGTGCGGGGAGACAAAACCTCGGGCGGCGGCGGCTGGGAAGACTTGAACT–3') from 49 bp to 108 bp downstream of the transcription initiation codon (Fig. 1). Analysis of the *PDK4* gene in the promoter region using peripheral blood indicated changes in the methylation rates as follows: CpG1 (8.2 ± 5.45% to 9.4 ± 1.65%, *P* = 0.50), CpG2 (7.1 ± 2.96% to 6.3 ± 2.53%, *P* = 0.89), CpG3 (6.6 ± 5.01% to 7.7 ± 1.96%, *P* = 0.50), CpG4 (7.6 ± 3.48% to 8.3 ± 1.52%, *P* = 0.50), CpG5 (6.2 ± 4.93% to 7.4 ± 1.55%, *P* = 0.50), and the average of the promoter region (CpG1-5 average) (7.2 ± 4.06% to 7.8 ± 1.48%, *P* = 0.50); these changes were not significantly different from preoperation to 5 months postoperation. Analysis of the *PDK4* gene in the promoter region in the skeletal muscle indicated the following changes: CpG1 (1.1 ± 1.06% to 2.2 ± 0.48%, *P* = 0.08), CpG2 (3.7 ± 1.26% to 3.3 ± 0.88%, *P* = 0.23), CpG3 (0.5 ± 1.10% to 0.3 ± 0.74%, *P* = 0.66), CpG4 (1.3 ± 2.24% to 1.7 ± 0.47%, *P* = 0.50), CpG5 (0.9 ± 2.05% to 1.3 ± 1.18%, *P* = 1.00), and the average of the promoter region (CpG1-5 average) (1.5 ± 1.44% to 1.8 ± 0.34%, *P* = 0.50); these changes were also not significant between preoperation to 5 months postoperation.

**Fig. 2:**
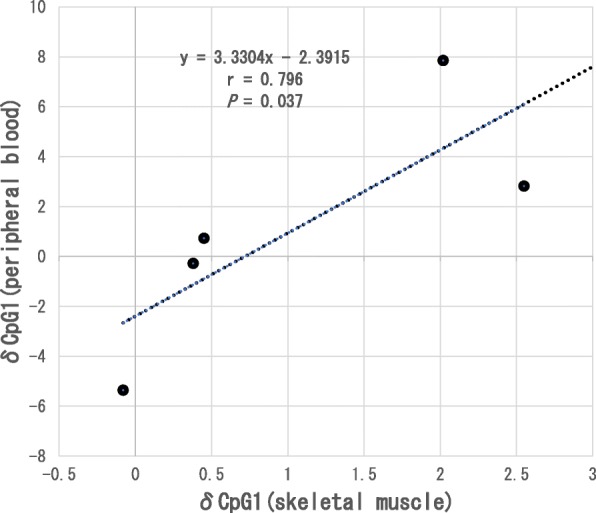
Correlation between the change in δCpG1 in the skeletal muscle and that in peripheral blood. Statistical analysis was performed using Spearman’s rank correlation coefficient

**Fig. 3:**
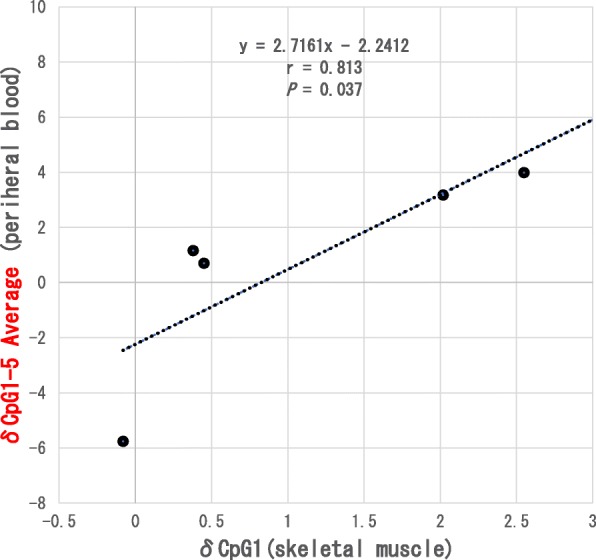
Correlation between changes in δCpG1 in the skeletal muscle and the δpromoter region in the peripheral blood. Statistical analysis was performed using Spearman’s rank correlation coefficient

However, as shown in Fig. 2, there was a significant positive correlation (*r* = 0.90, *P* = 0.04) between the change in methylation rate in δCpG1 in peripheral blood and that in the skeletal muscle. Furthermore, there was a significant positive correlation (*r* = 0.90, *P* = 0.04) between the change in the δCpG1 skeletal muscle methylation rate and the change in the methylation rate of the δpromoter region (CpG1-5 Average) in peripheral blood (Fig. 3). The difference in the amount of change between the skeletal muscle and peripheral blood was as follows: δCpG1 (skeletal muscle vs. peripheral blood) (1.2 ± 4.80% vs. 1.1 ± 1.15%, *P* = 0.89), δCpG2 (−0.9 ± 4.5% vs. −0.4 ± 0.58%, *P* = 0.89), δCpG3 (1.1 ± 4.7% vs. −0.16 ± 1.47%, *P* = 0.50), δCpG4 (0.72 ± 3.0% vs. 0.4 ± 2.26%, *P* = 0.89), δCpG5 (1.2 ± 4.4% vs. 0.4 ± 1.9%, *P* = 0.50), and average of δpromoter region (CpG1-5 Average) (0.7 ± 3.8% vs. 0.3 ± 1.19%, *P* = 0.69); these differences were not significantly different. In addition, there was no significant correlation between age and each position of δCpG1-5 nor between age and the δpromoter region (CpG1-5 Average) in peripheral blood and muscle.

## Discussion

Although we investigated epigenetic modifications related to postoperative exercise therapy using the skeletal muscle, such biopsies are painful and cause bleeding. If epigenetic modifications can be investigated via a blood test, the evaluation can be performed very easily and in a less invasive manner. However, the relationship between the skeletal muscle and blood is unclear. In CpG1, there was a significant positive correlation between the change in skeletal muscle methylation rate and the change in peripheral blood methylation rate. Furthermore, the change in the CpG1 skeletal muscle methylation rate was significantly positively correlated with the change in the average methylation rate of the promoter region in peripheral blood. Moreover, there was no significant difference in the amount of change between the skeletal muscle and peripheral blood. Based on the above, we speculated that CpG1 may be involved in methylation of the promoter region in the two tissues. Since *PDK4* is mainly present in the skeletal muscle and DNA methylation is highly tissue specific, it is common to collect and analyze the skeletal muscle [23]. However, Dayeh et al. reported that these epigenetic modifications altered by the physical and biological environment may affect all tissues of the body, and further, DNA methylation biomarkers in the blood may be used as surrogate markers for DNA methylation in inaccessible target tissues [24]. We also considered that lipid metabolism may affect the total fat mass via methylation of the *PDK4* gene and that evaluation with peripheral blood is possible. Therefore, it was considered clinically important to verify the methylation rate in both the skeletal muscle and peripheral blood tissues.

Our analysis revealed that methylation of CpG1 in the *PDK4* gene in the skeletal muscle might be associated with the average methylation rate of the total promoter region in the peripheral blood. However, its mechanism remains unclear because there are no studies that investigated the association between methylation differences in elderly skeletal muscle and peripheral blood samples. Pyrosequencing can be used to evaluate the methylation rate at each position in the same way as NGS [25], unlike conventional Sanger capillary sequencing [26, 27]. Pyrosequencing is different from NGS in that it can process a huge amount of data at high speed. The method is suitable for investigating CpG sites in a small region of DNA in a limited number of samples and genomic target regions and is considered to be cost-effective. As we investigated only the promoter region, we used pyrosequencing and revealed that CpG1 in the skeletal muscle *PDK4* gene might be associated with *PDK4* methylation in the peripheral blood.

We have previously reported the relationship between postoperative exercise therapy and body composition in elderly patients with OA knee by calculating the methylation rate of the *PDK4* gene, which controls the skeletal muscle and fat metabolism. We considered the possibility that the derived methylation rate could be one of the indicators of exercise therapy [21]. In postoperative exercise therapy, body composition did not change significantly based on DXA, and the promoter region of the *PDK4* gene in the skeletal muscle and peripheral blood did not change significantly. Barres et al. reported that exercise results in hypomethylation in the promoter region of the *PDK4* gene [16]. In this study, postoperative exercise therapy did not change the body composition of the patients. The reason may be that the exercise intensity could not be increased for these elderly patients, and the amount of exercise performed by each individual was small. Therefore, the exercise regimen may not have been sufficient to cause hypomethylation in the promoter region of the *PDK4* gene. In fact, body fat mass did not change significantly, and its influence is considered to be sufficient. Constantin et al. stated that the transcription of the *PDK4* gene inhibits PDC and suppresses the metabolism of pyruvate to acetyl-CoA [28]. It is possible that this process contributed to the reduction of body fat mass and weight loss through the activation of fatty acid oxidation by β-oxidation. If the body fat content decreased with body composition, it is possible that hypomethylation occurred in the promoter region.

There are several limitations to this study. First, the number of samples was small, and we did not include a control group. In this study, postoperative rehabilitation and exercise therapy were absolutely necessary treatments for all patients. Furthermore, it was extremely invasive and ethically impossible to perform similar tests, including muscle biopsy, on healthy individuals. In other words, it is possible that the change was only related to time or due to the dispersion of the measured value, and it cannot be denied that the result may change upon increasing the number of samples. Nonetheless, this is the first pilot study to investigate the methylation rate of each position of the CpG island in the promoter region of the *PDK4* gene as well as the correlation of the methylation rates between tissues. Therefore, we believe that these results are highly important, but large-scale research is needed to validate our findings. Second, we could perform exercise therapy for only 5 months due to the Japanese insurance system; in this period, we were unable to observe any changes in body composition during postoperative exercise therapy. Another limitation is that the subjects were elderly OA knee patients, and it is unclear whether similar outcomes would be obtained in young people. However, previous studies have reported that various genes are affected by methylation due to exercise in sedentary young people [16, 29, 30]. Fourth, methylation at positions other than the promoter region, i.e., in the non-promoter region, may be involved in *PDK4* gene transcription. Our previous study in patients who underwent exercise therapy after artificial knee arthroplasty also caused hypomethylation in the non-promoter region by exercising [20].. Pheiffer et al. reported that the non-promoter region has various transcriptional regulators [31]. However, the promoter region is most involved in transcription, and many previous studies used hypomethylation at the promoter region as a criterion; thus, it is appropriate for use in methylation analysis [16, 20, 29, 30]. Unlike NGS, pyrosequencing could not be used to comprehensively verify CpG islands, and therefore it was unclear whether the non-promoter region was involved. In addition, we could not evaluate mRNA, miRNA, or the protein involved in the expression of the *PDK4* gene. In the future, we plan to evaluate not only methylation but also histone modifications. Furthermore, by clarifying the mechanism behind protein expression of PDK4, it may be possible to understand fat metabolism mediated by the *PDK4* gene in more depth. Finally, methylation may also be affected by factors other than exercise. In this study, all patients received similar surgical procedures and rehabilitation, and pain relief was achieved; however, it was difficult to standardize dietary intake, and the only lifestyle intervention included exercise therapy. Nonetheless, the results of this study were likely attributable to the exercise therapy.

In this study, we utilized pyrosequencing as a cost-effective alternative to NGS; however, this method is still expensive and involves complicated procedures [32, 33]. Therefore, it is necessary to reduce costs and simplify procedures for widespread clinical application.

## Conclusion

The change in CpG1 methylation rate in the promoter region of *PDK4* in the skeletal muscle and in the peripheral blood following exercise therapy for 5 months postsurgery showed a significant positive correlation. Furthermore, the change in the methylation rate of the CpG1 region in the skeletal muscle showed a significant positive correlation with that of the average of the promoter region in the peripheral blood. These results suggested that the determination of the methylation rate of the *PDK4* gene by skeletal muscle analysis could be substituted by less invasive examination of the peripheral blood.

## Data Availability

The datasets used and analyzed during the current study are available from the corresponding author on reasonable request.

## Abbreviations

DXA: Dual-energy X-ray absorptiometry METs Metabolic equivalents NGS Next-generation sequencing OA knee Osteoarthritis of the knee PDC Pyruvate dehydrogenase complex RA Rheumatoid arthritis
